# SR-B1 and CD10 combined immunoprofile for differential diagnosis of metastatic clear cell renal cell carcinoma and clear cell carcinoma of the ovary

**DOI:** 10.1007/s10735-021-09963-3

**Published:** 2021-02-19

**Authors:** Teng Jiang, Xiaoli Diao, Meili Ding, Xiao Niu, Chao Wang, Yan Qi, Wei Jia, Lijuan Pang, Wenhao Hu, Hong Zou, Feng Li

**Affiliations:** 1 Department of Pathology, The First Affiliated Hospital, Shihezi University School of Medicine, Xinjiang, 832002 China; 2 Department of Pathology, Beijing Chao Yang Hospital, Capital Medical University, Beijing, 100020 China; 3 Department of Pathology, Ruijin Hospital, Shanghai Jiao Tong University School of Medicine, Shanghai, 200025 China

**Keywords:** Clear cell renal carcinoma, Clear cell carcinoma of the ovary, Immunohistochemistry, SR-B1, CD10

## Abstract

Both clear cell renal carcinoma (ccRCC) and clear cell carcinoma of the ovary (CCOC) have a clear cytoplasmic morphological feature, hence it is difficult to identify metastatic ccRCC and CCOC by morphology alone. At present, there are no effective immunohistochemical markers to distinguish between these two tumors. Studies have shown that the clear cytoplasm of ccRCC is mainly caused by cholesterol-rich lipids in the cytoplasm, while that of CCOC is due to the accumulation of cytoplasmic glycogen. Objective: to hypothesize that the scavenger receptor class B-type 1 (SR-B1) protein responsible for HDL cholesterol uptake may be differentially expressed in ccRCC and CCOC, and high CD10 expression in the renal tubular epithelium may assist in distinguishing between ccRCC and CCOC. Methods: effective immunohistochemical markers were applied in 90 cases of renal clear cell carcinoma and 31 cases of ovarian cancer to distinguish between the two types of tumors.Result: SR-B1 and CD10 expression is significantly higher in ccRCC than CCOC. Both SR-B1 and CD10 exhibited focal weak-medium intensity staining in CCOC, and their staining extent and intensity were significantly lower than ccRCC. The sensitivity and specificity of SR-B1 for identifying ccRCC were 74.4% and 83.9%, respectively. The sensitivity and specificity of CD10 for identifying CCOC were 93.3% and 80.6%, respectively. The combined SR-B1( +) CD10( +) immunoprofile supports the diagnosis of ccRCC with a specificity of 93.5%. The combined SR-B1(-) CD10(-) immunoprofile supports the diagnosis of CCOC with a specificity of 93.3%. Conclusions: our findings demonstrate that the combination of SR-B1 and CD10 immunoprofiling is a valuable tool for differential diagnosis of ccRCC and CCOC.

## Introduction

Clear cell renal cell carcinoma (ccRCC) is the most common malignant tumor of the renal parenchyma. The incidence of ccRCC is increasing every year with an average annual rate of 2%, and approximately 20% to 30% of patients with ccRCC have recurrence or metastasis within 3 years after surgery (NCCN Guidelines® 2019). Clear cell carcinoma of the ovary (CCOC) is characterized by resistance to conventional chemotherapy, early recurrence, and a poor prognosis (Gulec et al. 2015; Sugiyama et al. 2000) CCOC and ccRCC share morphological similarities such as a clear cytoplasm, and tubular, flaky or papillary structures. Moreover both tumors express PAX8, a transcription factor normally expressed by cells of the nephric, thyroid, and Mullerian duct lineage (Köbel et al. 2016) Consequently, distinguishing between CCOC and metastatic ccRCC to the ovary based on histomorphology alone is challenging. Thus far, there is a lack of effective immunohistochemical markers for differentiating these two tumors. Recent studies have shown that CCOC and ccRCC show different metabolism characteristics. The cytoplasmic transparency of ccRCC is mainly due to the large amount of cytoplasmic cholesterol ester (Gebhard et al. 1987; Tosi et al. 2004), while the clear cytoplasm of CCOC is more likely due to the accumulation of cytoplasmic glycogen (Tavassoli and Devilee 2003), suggesting that different energy metabolism characteristics may assist in a differential diagnosis.

Scavenger receptor class B type 1 (SR-B1), also known as HDL receptor, is a glycoprotein located on the surface of cell membranes, and the only molecule that acts on the selective absorption of HDL cholesterol ester. Our previous studies have found that SR-B1 is highly expressed in ccRCC and promotes tumor proliferation and invasion; however, the expression of SR-B1 in CCOC has not been examined. In addition, CD10, a marker of the renal tubular epithelium is highly expressed in ccRCC (Fletcher et al. 2013), but the expression of CD10 in CCOC has not been determined. We hypothesize that the SR-B1 protein is differentially expressed in ccRCC and CCOC, and a combined immunoprofiling of SR-B1 and CD10 will assist in the differential diagnosis of these two cancers.

## Materials and methods

### Tumor specimens

The 90 cases of ccRCC (collected from 1974 to 2013) used in this study were provided by the Department of Pathology, the First Affiliated Hospital of Shihezi University School of Medicine. The 31 cases of CCOC were provided by the Department of Pathology, Beijing Chao Yang Hospital of Capital Medical University. All patients had complete medical history and clinical pathological data and all cases were confirmed by surgery and pathology.

### Immunohistochemistry

Paraffin blocks and corresponding hematoxylin and eosin (HE) stained sections were collected, and the HE sections were evaluated by two senior pathologists (HZ and FL). Biopsy specimens were fixed in 10% neutral-buffered formalin and routinely processed. Paraffin-embedded blocks were sectioned (5-μm thick), stained with HE and observed by microscopy (Olympus, CX23). The two-step immunohistochemical envision method was applied. To confirm the specificity of the immunoreaction, known positive and negative tissues were used as controls. The intensity of staining was evaluated for each marker and assigned an incremental score of 0, 1 + , 2 + , and 3 + . The extent of staining was categorized as focal (< 25%), multifocal (25% to 75%), or diffuse (> 75%).

### Statistical analysis

The sensitivity and specificity of each marker and the diagnostic positive predictive values of their combination were subsequently determined in both types of tumor. The difference between the two groups was compared by the chi-square test.

All statistical analyses was performed using the SPSS version 17.0, and *p*-values < 0.05 were considered significant. The sensitivity, specificity, and positive predictive values were calculated by evaluation of the screening and diagnostic tests.

## Results

### Clinical features

The average age of the 90 patients with ccRCC was 58.5 (28 to 77) years old, including 63 males and 27 females with an average age of 60.1 (39 to 76) years. All 31 patients with CCOC were female patients with an average age of 52.3 (32 to 75) years.

### Pathological findings

**Fig. 1:**
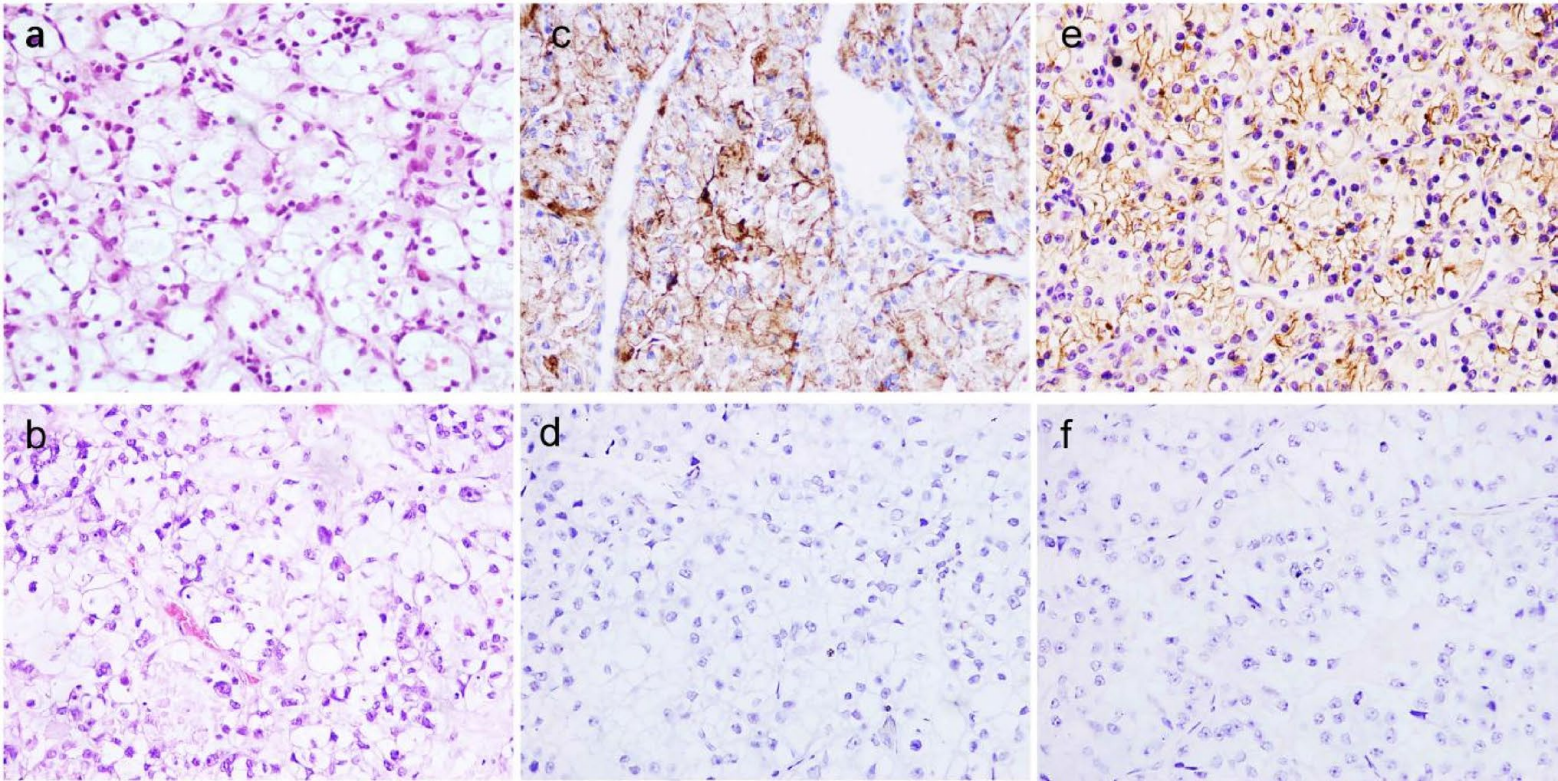
Both ccRCC (**a**) and CCOC (**b**) exhibit a clear cytoplasmic morphological feature, × 200 magnification. SR-B1 in ccRCC show a strong positive expression in the cytoplasm and membrane (**c**) but is negatively or weakly expressed in CCOC (**d**). CD10 expression is strongly positive in the cytoplasm and membrane (**e**) in ccRCC but is negatively or weakly expressed in CCOC (f), × 200 magnification

In all 90 cases of ccRCC, nest-sheet and acinar cells were mainly arranged (Fig. 1a), and some were microencapsulated, papillary, papillary-tubular and glandular-tubular. Most of the tumor cells exhibited a clear cytoplasm, with a few eosinophils and abundant blood vessels in the stroma. Cell morphology included round, multilateral, or cubic. The cytoplasm was transparent and eosinophilic. The cells of the 31 CCOC cases were solid, papillary, nested, and transparent. Sixteen of the 31 (51.6%) cases had typical spike-like cells (Fig. 1b).

### Immunohistochemistry

Of the 90 ccRCC cases, 67 (74.4%) demonstrated membranous and cytoplasmic SR-B1 positivity that was diffuse in 24 cases and multifocal in 43 cases. The staining intensity scores ranged from 2 + to 3 + . The staining intensity of SR-B1 was higher in nuclear high-grade ccRCC (Fig. 1c). Only 5 (16.1%) of the 31 CCOC cases were SR-B1 focal positive (Fig. 1d) with a staining intensity score of 1 + . Eighty four (93.3%) of the 90 ccRCC cases exhibited membranous CD10 positivity (Fig. 1e) that was diffuse in 53 cases and multifocal in 31 cases. The intensity of staining ranged from 2 + to 3 + (Table 1). Membranous CD10 expression was present in 6 of 31 (19.4%) evaluable CCOC cases (Fig. 1f), and the intensity of staining was 1 + . Sixty-seven ccRCC cases were positive for both SR-B1 and CD10. Two cases of CCOC were positive for both SR-B1 and CD10. Five cases of SR-B1 positivity and 4 cases of CD10 positivity in CCOC showed shoe-nail-like cells. Two cases of CD10 positivity in CCOC were clear cells. The remaining cases that lacked typical nail-like cells and were clear were negative for both SR-B1 and CD10 (Table 2).

**Table 1:** Antibodies used in this study

Marker	Source	Type	Dilution	Antigen retrieval
SR-B1	ABCAM	ab52629 MAb	1:200	Yes (citrate)
CD10	ZSGB-BIO	3410 MAb	1:100	Yes (citrate)

**Table 2:** Intensity and extent of immunohistochemical staining of SR-B1 and CD10 in CCOC and ccRCC

Antibody	Intensity category (Extent)	CCRCC		CCOC	
		Intensity	Extent	Intensity	Extent
SR-B1	Negative	23 (25.6)	23 (25.6)	26 (83.9)	26 (83.9)
	1 (Focal)	0 (0)	0 (0)	5 (16.1)	5 (16.1)
	2 (Multifocal)	43 (47.8)	43 (47.8)	0 (0)	0 (0)
	3 (Diffuse)	24 (26.6)	24 (26.6)	0 (0)	0 (0)
CD10	Negative	6 (6.7)	6 (6.7)	25 (80.6)	25 (80.6)
	1 (Focal)	0 (0)	0 (0)	6 (19.4)	6 (19.4)
	2 (Multifocal)	31 (34.4)	31 (34.4)	0 (0)	0 (0)
	3 (Diffuse)	53 (58.9)	53 (58.9)	0 (0)	0 (0)

SR-B1 and CD10 in CCOC were focal positive with a significantly lower staining extent and intensity than ccRCC. SR-B1 and CD10 expression was significantly higher in ccRCC than CCOC (p < 0.01). SR-B1 positivity supported the diagnosis of ccRCC with a sensitivity of 74.4% and a specificity of 83.9%. CD10 positivity supported the diagnosis of CCOC with a sensitivity of 93.3% and a specificity of 80.6% (Table 3). Combined SR-B1 and CD10 immunopositivity supported the diagnosis of ccRCC with a sensitivity of 74.8%, specificity of 93.5%, and positive predictive value of 97.1%. Combined SR-B1 and CD10 immunonegativity supported the diagnosis of CCOC with a sensitivity of 71.0%, specificity of 93.3%, and positive predictive value of 78.6% (Table 4).

**Table 3:** Immunohistochemical expression of SR-B1 and CD10 in ccRCC and CCOC

	SR-B1		CD10	
Tumor type	Positive (%)	Negative (%)	Positive (%)	Negative (%)
ccRCC	67/90 (74.4)	23/90 (25.6)	84/90 (93.3)	5/90 (6.7)
CCOC	5/31 (16.1)	26/31 (83.9)	6/31 (19.4)	25/31 (80.6)

**Table 4:** Sensitivity and specificity of SR-B1 and CD10 and SR-B1 or CD10 immunostaining in the diagnosis of CCOC versus ccRCC

Test	Sensitivity (%)	Specificity (%)	PPV (%)
SR-B1 for ccRCC	74.4	83.9	93.1
CD10 for ccRCC	93.3	80.6	93.3
SR-B1 (+) and CD10 (+) for ccRCC	74.4	93.5	97.1
SR-B1 (−) and CD10 (−) for CCOC	71.0	93.3	78.6

## Discussion

**Fig. 2:**
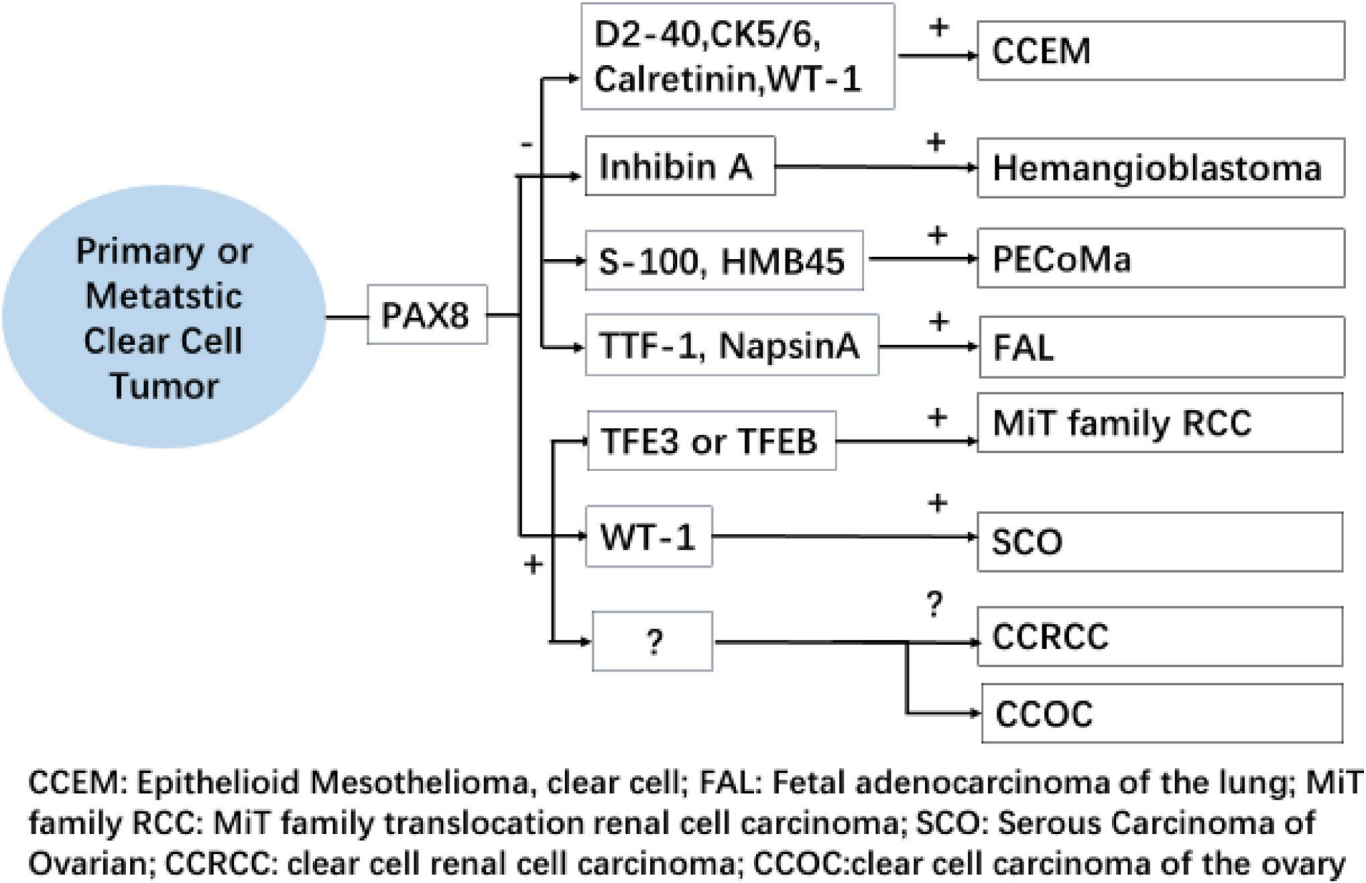
Immunohistochemical map for the identification of common clear cell tumors; ccRCC and CCOC lack effective immunohistochemical markers for differential diagnosis

In addition to ccRCC and CCOC, other common cytoplasmic tumors include Perivascular epithelioid cell tumor (PEComa), MiT family translocation renal cell carcinoma, fetal adenocarcinoma of the lung, hemangioblastoma, clear cell malignant mesothelioma, and serous carcinoma of the ovary. A differential diagnosis is needed when metastasis occurs, but the latter has relatively specific immunohistochemical markers to assist in diagnosis (Fig. 2), such as melanin labeling S-100 and HMB45 positivity in PEComa (Fletcher et al. 2013), TFE3 or TFEB positivity in MiT family translocation renal cell carcinoma (Moch et al. 2016), TTF-1 and NapsinA positivity in fetal adenocarcinoma of the lung (Huang and Chen 2016), Inhibin A positivity and PAX8/PAX2 negativity in hemangioblastoma (Carney et al. 2011), D2-40, CK5/6, Calretinin and WT-1 positivity in malignant mesothelioma (Travis et al. 2015), and PAX8 and WT-1 positivity in serous carcinoma of the ovary (Kuhn and Ayhan 2018). In ccRCC and CCOC, PAX-8 positivity is difficult to differentiate if metastasis occurs. In addition, ccRCC is well known for the tendency to metastasize to organs including the lung, bone, and ovary. In this study, only about 50% of CCOC have characteristic shoe-nail-like clear cells. The other common structures are nested, aciniform, microcapsule, papillary, nipple-tubular, and glandular cells, which are similar to the morphology and structure of ccRCC. Since ccRCC and CCOC have different prognosis and treatment schemes, immunohistochemistry is particularly important as a diagnostic supplement when histological morphology alone cannot differentiate between the two carcinomas. Therefore, it is necessary to find immunohistochemical markers with high sensitivity and specificity for diagnosing ccRCC and CCOC.

**Fig. 3:**
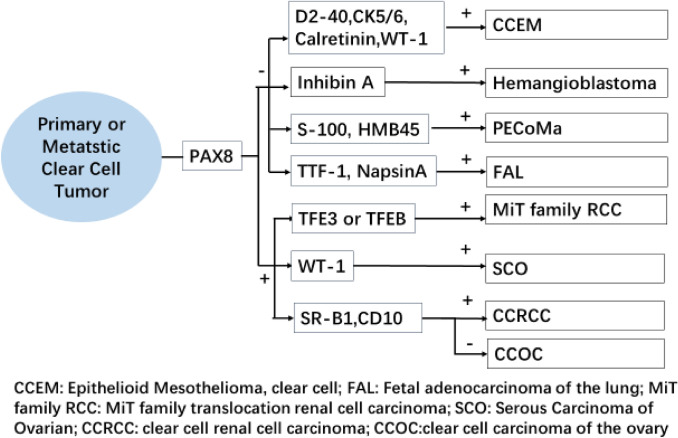
Immunohistochemical map for the identification of common clear cell tumors. SR-B1 combined with CD10 can help in the differential diagnosis of ccRCC and CCOC

The difficulty in identifying the primary site is a major problem in diagnosing female reproductive system tumors, and highlights the need to improve the diagnosis rate of primary CCOC. PAX8 (Köbel et al. 2016) is emerging as the most specific marker for distinguishing between primary and metastatic ovarian tumors. However, PAX8 can be expressed in both ccRCC and CCOC, which makes it difficult to determine the primary site of the tumor by PAX8 positivity. Some studies have found that HNF-1β, WT-1, and NapsinA are highly expressed in CCOC, but lack specificity or sensitivity. These markers can also be expressed in varying degrees in ccRCC (Ji et al. 2018), which makes their application a challenge. Recently a few studies have reported on the expression of CD10 in CCOC and its value in distinguishing between ccRCC and CCOC. The present study, based on the perspective of tumor metabolism, found that expression of the SR-B1 protein and CD10 was significantly higher in ccRCC than CCOC. SR-B1 and CD10 exhibited focal weak-medium intensity staining in CCOC with a staining range and intensity that is significantly lower than ccRCC. The specificity of SR-B1 for ccRCC diagnosis was 83.9% and the specificity of CD10 for CCOC diagnosis was 80.6%. When SR-B1 and CD10 immunopositivity were combined, specificity of the ccRCC diagnosis was increased to 93.5%. When both SR-B1 and CD10 were negative, the specificity of the CCOC diagnosis was increased to 93.3%. Taken together, our results show that SR-B1 and CD10 immunoreactivity have higher sensitivity and specificity for ccRCC diagnosis, and can improve specificity when combined, even for identifying tumors with clear cell morphology (Fig. 3).

In conclusion, our study confirms that SR-B1 and CD10 are highly expressed in ccRCC but only weakly expressed or not expressed at all in CCOC. The combination of SR-B1 and CD10 can be used as new markers in the differential diagnosis of ccRCC and CCOC. This will help clinicians and pathologists identify the primary lesions of tumors and adopt appropriate treatments to avoid misdiagnosis and mistreatment.
